# Supplementary material for: Modelling donor factors influencing pancreas transplant utilization and evolution of decision-making over time
Source: Commun Med (Lond). 2026 Mar 7;6:231. doi: 10.1038/s43856-026-01506-9 (PMC13096168; doi:10.1038/s43856-026-01506-9)

Supplementary material

Supplementary Tables

Supplementary Table 1: Comparison of coefficients (95% CI) for donor variables between logistic regression model without OPO term and mixed-effects model with random intercept for OPO (to account for clustering, and utilization differences between OPOs). Variables marked with apostrophes (′ and ′′) represent the restricted cubic spline terms used to model non-linear relationships. Plots for these variables are shown in Supplementary Figure 2. Abbreviations: CMV = cytomegalovirus, DCD = donation after circulatory death, IV = intravenous, HLA = human leukocyte antigen, BMI = body mass index, ALT = alanine aminotransferase, OPO = organ procurement organisation.

Variable	Coefficient (95% CI) from logistic regression model without OPO term	Coefficient (95% CI) from mixed effects model with random intercept for OPO
BMI	0.075 (0.061-0.09)	0.076 (0.062-0.091)
BMI′	-0.647 (-0.72--0.573)	-0.671 (-0.746--0.596)
BMI′′	1.175 (0.895-1.454)	1.232 (0.948-1.517)
Age	0.064 (0.058-0.069)	0.065 (0.06-0.071)
Age′	-0.411 (-0.431--0.39)	-0.424 (-0.445--0.403)
Age′′	1.009 (0.891-1.126)	1.048 (0.929-1.167)
Donation date	-0.057 (-0.069--0.045)	-0.059 (-0.071--0.046)
Donation date′	0.037 (0.023-0.052)	0.039 (0.024-0.054)
Ethnicity	-0.037 (-0.055--0.02)	-0.029 (-0.049--0.01)
Sex	0.003 (-0.046-0.051)	-0.006 (-0.055-0.043)
CMV	-0.09 (-0.134--0.046)	-0.039 (-0.085-0.006)
Blood group	0.062 (0.046-0.077)	0.065 (0.049-0.08)
Cause of death	0.008 (-0.01-0.027)	0.011 (-0.008-0.03)
DCD donor	-2.557 (-2.665--2.449)	-2.653 (-2.763--2.543)
Given insulin 24 hours before cross-clamp	-0.082 (-0.126--0.038)	-0.078 (-0.127--0.029)
Heavy alcohol use	-0.562 (-0.636--0.489)	-0.585 (-0.66--0.51)
Coronary artery disease	-0.934 (-1.431--0.436)	-0.929 (-1.428--0.43)
Smoking	-0.171 (-0.271--0.071)	-0.173 (-0.274--0.071)
Hypertension	-0.531 (-0.628--0.435)	-0.52 (-0.617--0.422)
IV drug use	-0.546 (-0.629--0.463)	-0.584 (-0.668--0.499)
Latest blood pH	4.17 (3.229-5.111)	4.366 (3.403-5.329)
Latest blood pH′	-3.183 (-4.852--1.513)	-3.06 (-4.765--1.355)
Latest blood pH′′	7.469 (-4.658-19.596)	5.549 (-6.82-17.917)
Hepatitis C	-1.8 (-1.955--1.646)	-1.871 (-2.028--1.714)
Number of rare HLA variants	-0.041 (-0.071--0.01)	-0.029 (-0.062-0.003)
Peak lipase	0.001 (-0.001-0.004)	-0.001 (-0.003-0.002)
Peak lipase′	-0.219 (-0.416--0.021)	-0.11 (-0.313-0.092)
Peak lipase′′	0.311 (0.033-0.588)	0.16 (-0.124-0.444)
Peak ALT	-0.007 (-0.008--0.005)	-0.007 (-0.009--0.006)
Peak ALT′	0.711 (0.521-0.902)	0.791 (0.596-0.985)
Peak ALT′′	-0.954 (-1.211--0.697)	-1.06 (-1.323--0.798)
Peak creatinine	1.624 (1.406-1.842)	1.724 (1.502-1.946)
Peak creatinine′	-15.091 (-16.747--13.436)	-15.617 (-17.304--13.93)
Peak creatinine′′	36.707 (32.043-41.372)	37.809 (33.058-42.561)
Admission length	0.1 (0.054-0.146)	0.106 (0.059-0.153)
Admission length′	-1.699 (-2.548--0.85)	-1.641 (-2.508--0.773)
Admission length′′	2.563 (1.244-3.883)	2.454 (1.106-3.801)
Inotropic support used	-0.032 (-0.076-0.012)	-0.062 (-0.109--0.015)
OPO impact		P<0.001

Supplementary Table 2: Multivariable logistic regression sensitivity analysis for pancreas utilisation for the full cohort (n=133986). P-values are from two-sided Wald-tests. An event was defined as successful pancreas transplant (n=14612). This sensitivity analysis includes amylase and AST, which were excluded in the main analysis due to collinearity with lipase and ALT respectively. HbA1c is also included in this sensitivity analysis due to being excluded in the main analysis for having substantial missing data. For restricted cubic spline terms, a p-value is given for the overall impact of the variable on outcome (Wald-test); plots for these restricted cubic splines are shown in Supplementary Figure 3. Abbreviations: CMV = cytomegalovirus, DCD = donation after circulatory death, IV = intravenous, HLA = human leukocyte antigen, BMI = body mass index, AST = aspartate aminotransferase.

**Donor variable**	**Adjusted Odds Ratio (95% CI)**	**P-value**
Ethnicity		
White	Ref	
Asian, Non-Hispanic	1.099 (0.945 to 1.278)	0.220
Black, Non-Hispanic	1.205 (1.135 to 1.280)	<0.001
Hispanic/Latino	0.847 (0.797 to 0.901)	<0.001
Other	0.731 (0.605 to 0.883)	0.001
Sex: male	1.001 (0.953 to 1.051)	0.963
CMV positive	0.913 (0.873 to 0.955)	<0.001
Blood group		
A	Ref	
AB	0.284 (0.244 to 0.331)	<0.001
B	0.922 (0.859 to 0.991)	0.027
O	1.153 (1.101 to 1.208)	<0.001
Cause of death		
Anoxia	Ref	
Cerebrovascular/stroke	0.972 (0.891 to 1.059)	0.513
Drug overdose	1.084 (0.998 to 1.178)	0.055
Head trauma	1.059 (0.997 to 1.125)	0.064
Other	0.730 (0.633 to 0.842)	<0.001
DCD donor	0.078 (0.070 to 0.087)	<0.001
Given insulin 24 hours before cross-clamp	0.963 (0.921 to 1.007)	0.098
Heavy alcohol use	0.566 (0.526 to 0.610)	<0.001
Coronary artery disease	0.391 (0.238 to 0.643)	<0.001
Smoking	0.849 (0.768 to 0.939)	0.001
Hypertension	0.584 (0.530 to 0.644)	<0.001
IV drug use	0.582 (0.533 to 0.637)	<0.001
Hepatitis C antibody positive	0.163 (0.140 to 0.191)	<0.001
Number of rare HLA variants		
0	Ref	
1	0.961 (0.907 to 1.018)	0.176
2	0.857 (0.718 to 1.022)	0.085
3	0.711 (0.485 to 1.041)	0.079
4	0.795 (0.453 to 1.396)	0.425
Inotropic support used	0.983 (0.940 to 1.028)	0.454
RCS: BMI	RCS terms	<0.001
RCS: Age, years	RCS terms	<0.001
RCS: Donation date	RCS terms	<0.001
RCS: Latest blood pH	RCS terms	<0.001
RCS: Peak amylase	RCS terms	<0.001
RCS: Peak AST	RCS terms	<0.001
RCS: Peak creatinine	RCS terms	<0.001
RCS: Hospital stay, days	RCS terms	<0.001
RCS: HbA1c	RCS terms	<0.001

Supplementary Figures

Supplementary Figure 1: Absolute number of pancreas transplants per year from 2010 to 2023.

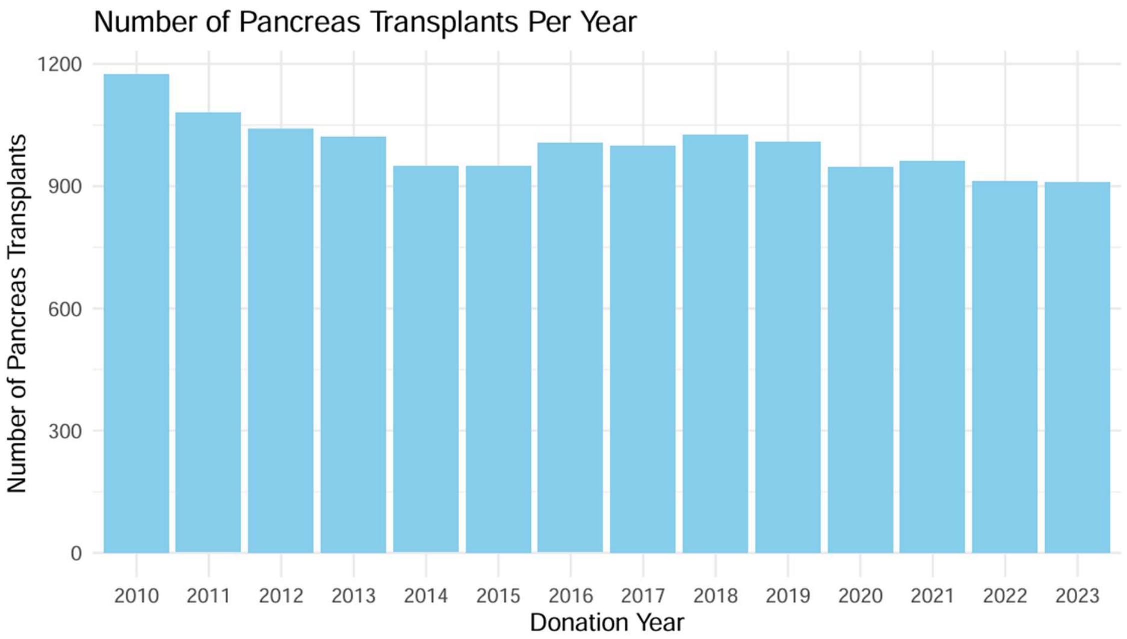

Supplementary Figure 2: Sensitivity analysis accounting for organ procurement organization (OPO) effect. Restricted cubic spline models showing associations between key continuous variables and pancreas utilisation from a mixed-effects logistic regression model with random intercept for OPO (Supplementary Table 1). Restricted cubic splines were plotted using 4 knots. The solid line represents the estimated odds ratio (OR) for pancreas utilisation relative to the average value of each variable, with grey shading indicating the 95% confidence interval. The dotted horizontal line at OR=1 represents no effects on utilisation relative to the reference value. The green shaded area indicates association with increased utilisation, and the red shaded area indicates association with decreased utilisation. Donor factors displayed are: A) Age. B) Peak ALT. C) BMI. D) Peak creatinine. E) Donation date. F) Peak lipase. A log-scaled y-axis is used for all variables for better visualisation of relationships. A log-scaled x-axis is also used for peak lipase, peak ALT and peak creatinine.

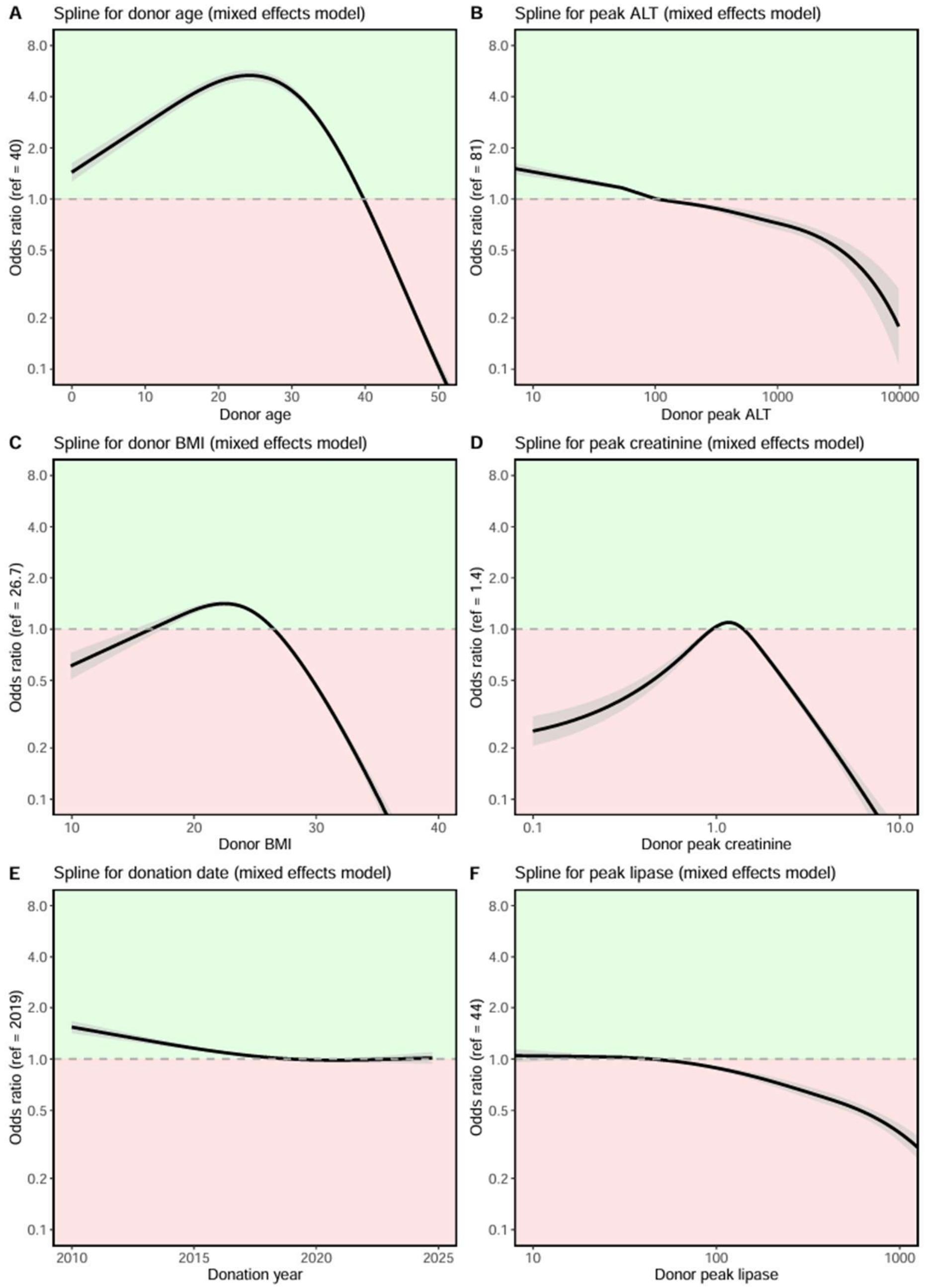

Supplementary Figure 3: Restricted cubic spline models showing association between donor variables from sensitivity analysis and pancreas utilisation. Sensitivity analysis includes amylase and AST, which were excluded due to collinearity with lipase and ALT respectively. HbA1c is also included in the sensitivity analysis and not the main analysis due to substantial missing data. Restricted cubic splines were plotted using 4 knots and were adjusted for all variables in Supplementary Table 2. The solid line represents the estimated odds ratio (OR) for pancreas utilisation relative to the average value of each variable, with grey shading indicating the 95% confidence interval. The dotted horizontal line at OR=1 represents no effects on utilisation relative to the reference value. The green shaded area indicates association with increased utilisation, and the red shaded area indicates association with decreased utilisation. A log-scaled y-axis is used for all variables for better visualisation of relationships. A log-scaled x-axis is also used for peak amylase, peak AST and peak creatinine. A) BMI. B) Age. C) Donation date. D) Latest blood pH. E) Peak amylase. F) Peak AST. G) Peak creatinine H) Hospital stay length. I) Peak HbA1c.

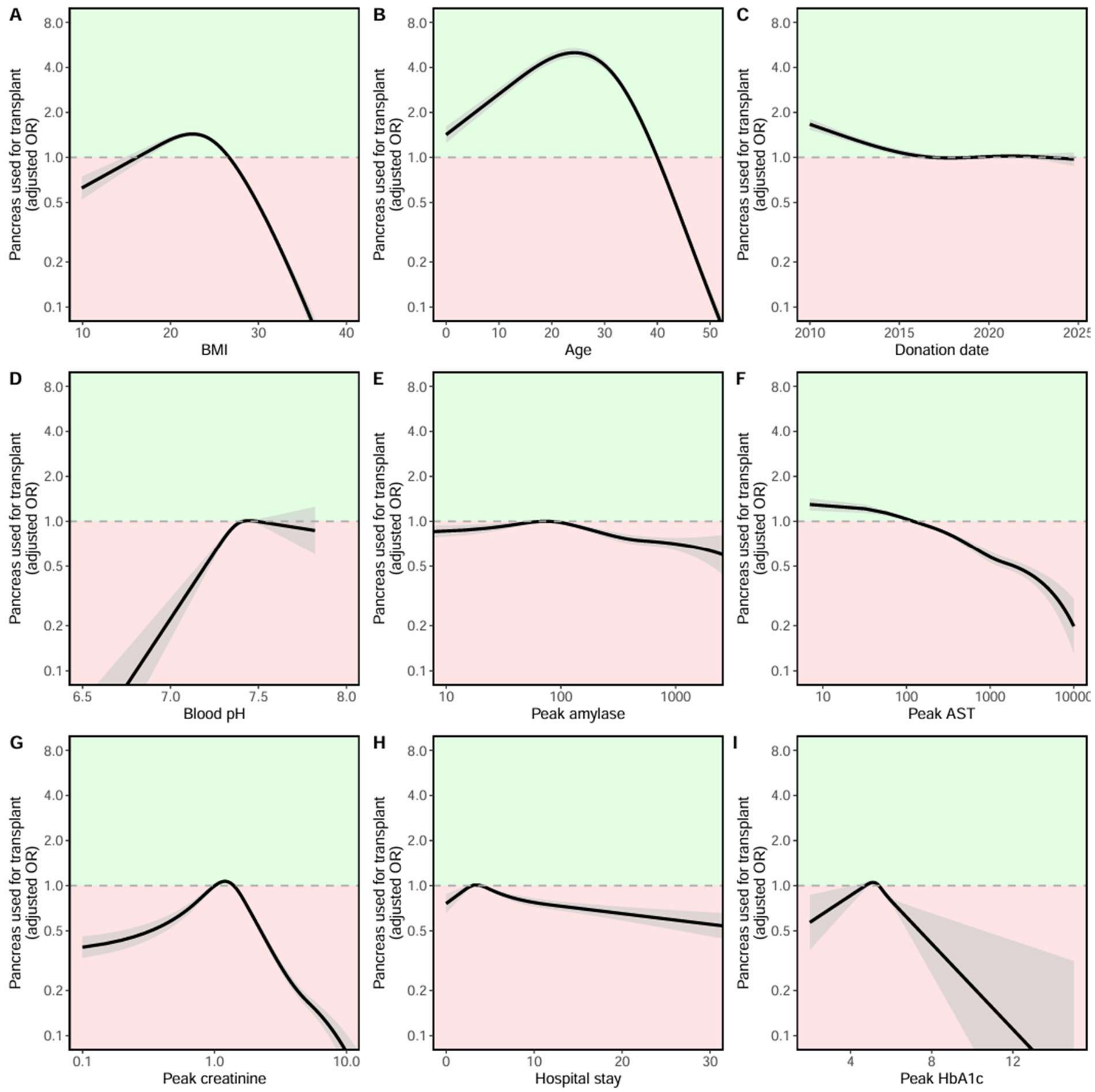

Supplementary Figure 4: Plot showing Wald chi-square statistics for variables from main analysis using ANOVA function from rms package. Values further right represent greater importance of the variable in determining utilisation decisions, with age being the most important, and sex being the least important factor.

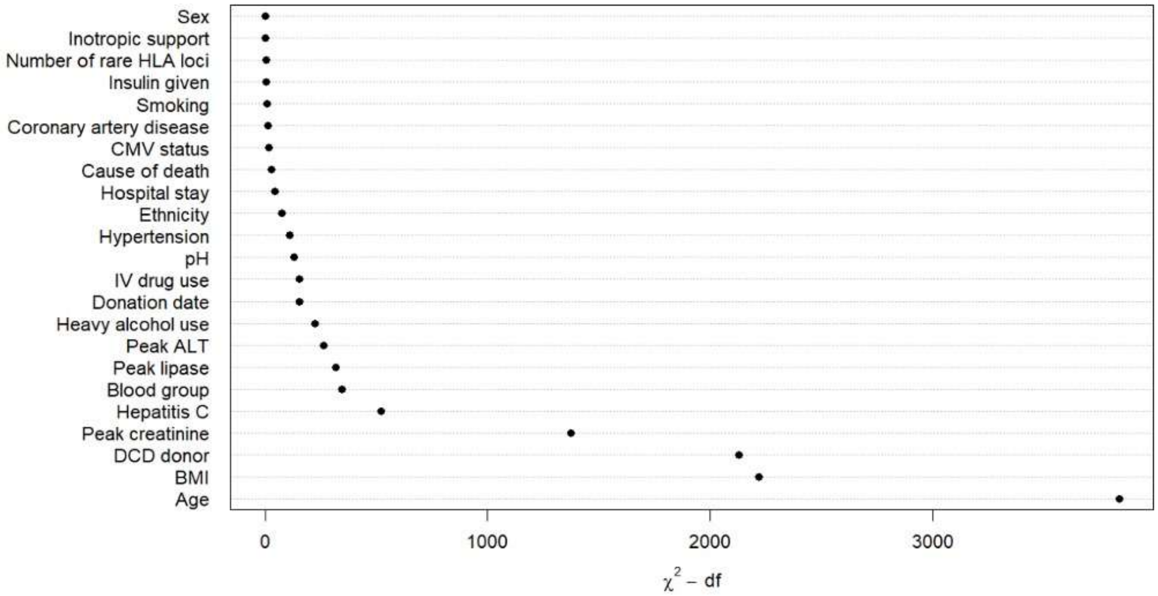

Supplementary Figure 5: Restricted cubic spline models showing interaction effects between variables and donation date on pancreas utilisation. Grey shading indicates the 95% confidence interval. Restricted cubic splines were plotted using 3 knots with a log scaled y-axis. Interaction models for key variables are shown in Figure 3. A) Donor blood group. B) Donor heavy alcohol use. C) Donor BMI. D) Donor peak creatinine. E) Donor peak lipase. F) Donor peak ALT

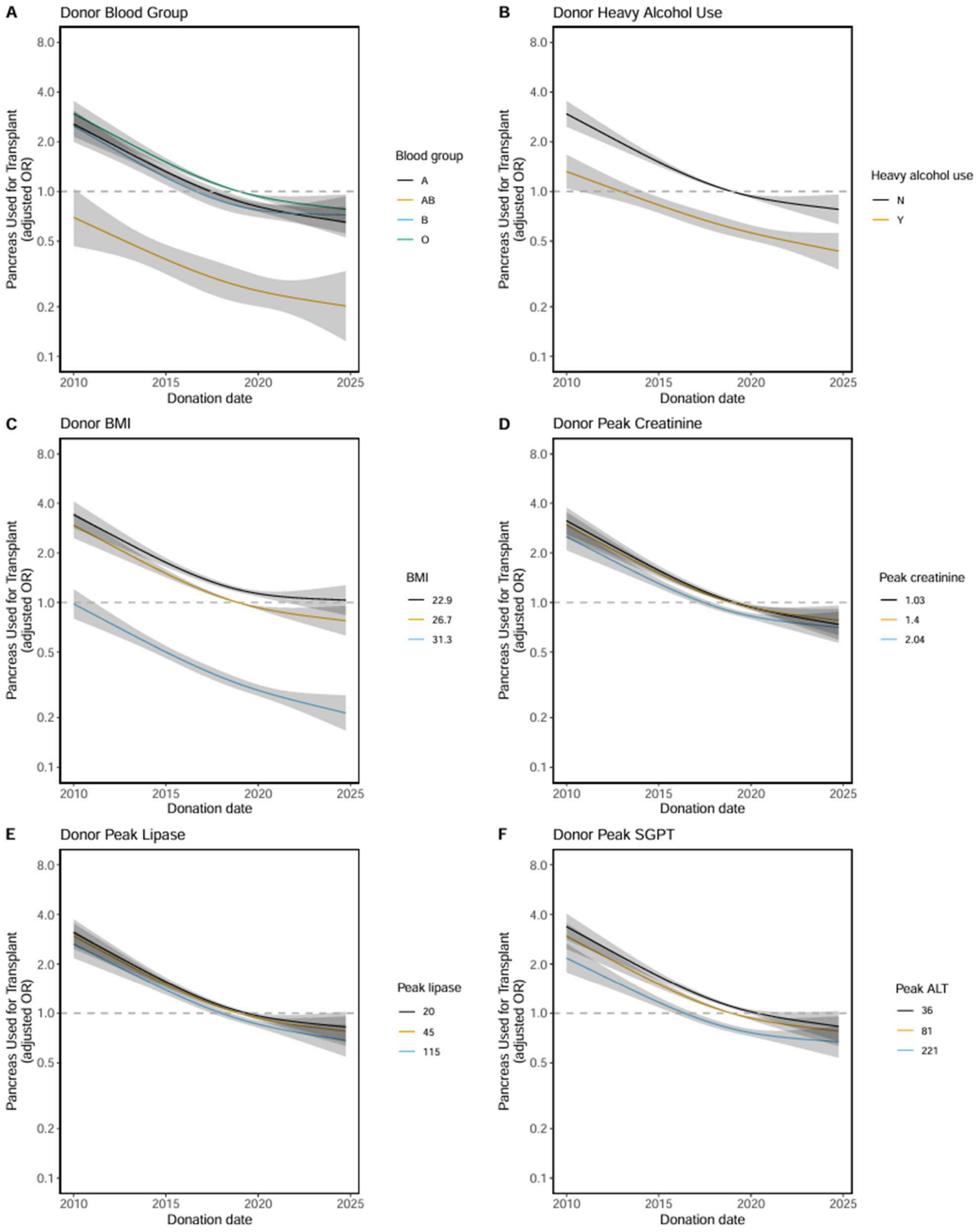

Supplementary Figure 6: Plot of centre-level DCD transplant percentage by total transplant volume over the study period. Blue line represents a LOESS smoothed trend with 95% confidence interval (grey band). Centres performing less than 5 pancreas transplants per year were excluded as DCD utilisation percentage would be too unstable for comparison.

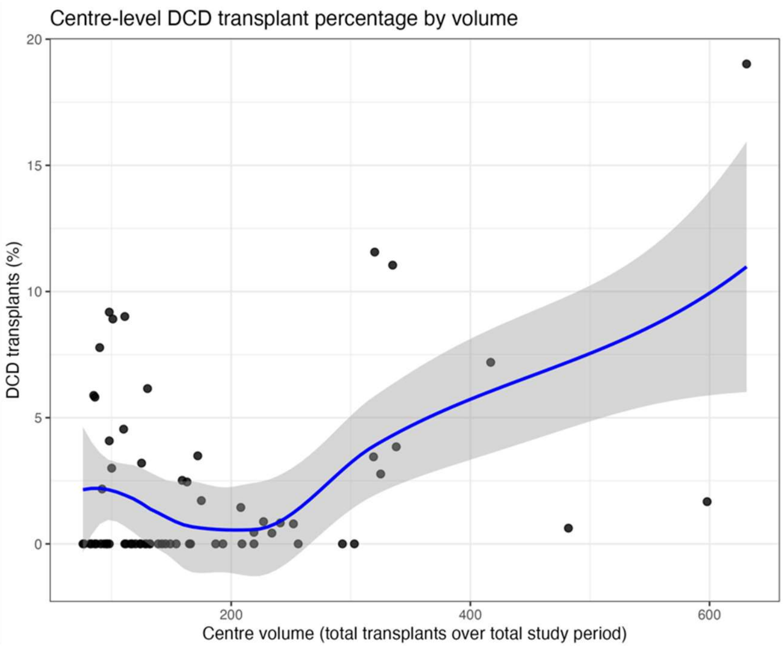

Supplementary Figure 7: Counts of annual DCD donors by retrieval type over the study period. A) DCD donors stratified by retrieval type in the full cohort. B) DCD donors stratified by retrieval type in those utilised for pancreas transplantation. Abbreviations: SRR = super rapid recovery, NRP = normothermic regional perfusion.

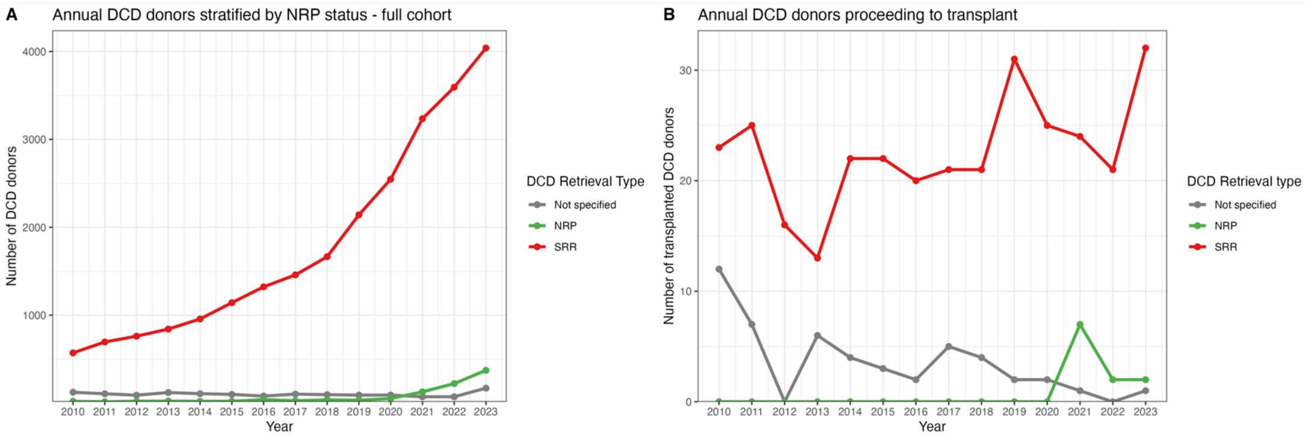

Supplementary Figure 8: Breakdown of HCV antibody positive donors by HCV NAT status. A&B) HCV antibody positive donors in the full cohort (including those donors where the pancreas was not transplanted). C&D) HCV antibody positive donors where the pancreas was transplanted. Counts (panel A&C) and proportions (B&D) are displayed. Proportions are only displayed from 2017 onwards, as prior to this too few HCV antibody positive donor pancreas transplants took place.

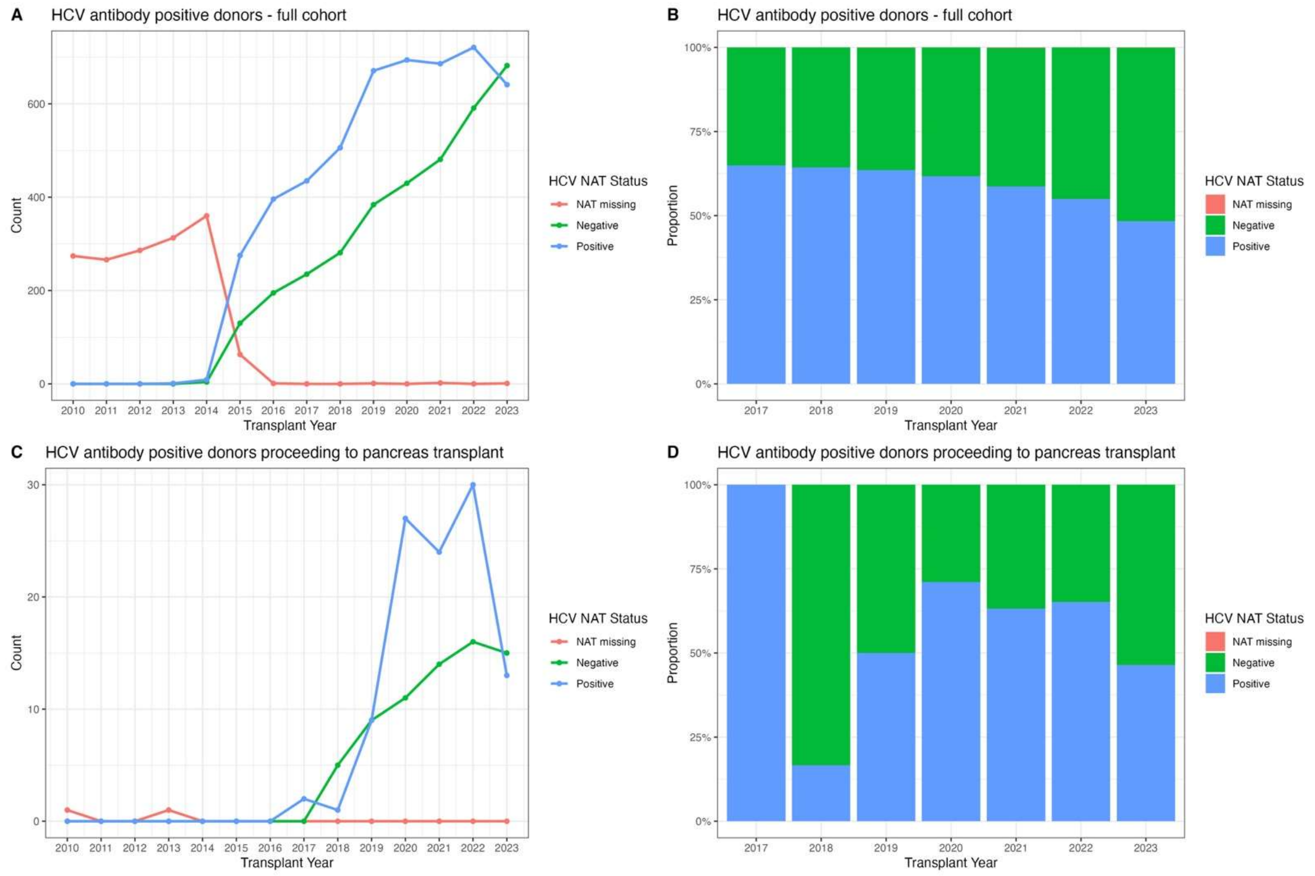

Supplementary Figure 9: Counts of key donor factors in the full cohort (donors where at least one organ was retrieved, including those donors where the pancreas was not transplanted) over the study period. A) Age group. B) IV drug use. C) Donor type. D) Hepatitis C status. E) BMI. 'NA' represents donors with missing data for the relevant variable. Abbreviations: IVDU = intravenous drug use, DCD = donation after circulatory death, BMI = body mass index.

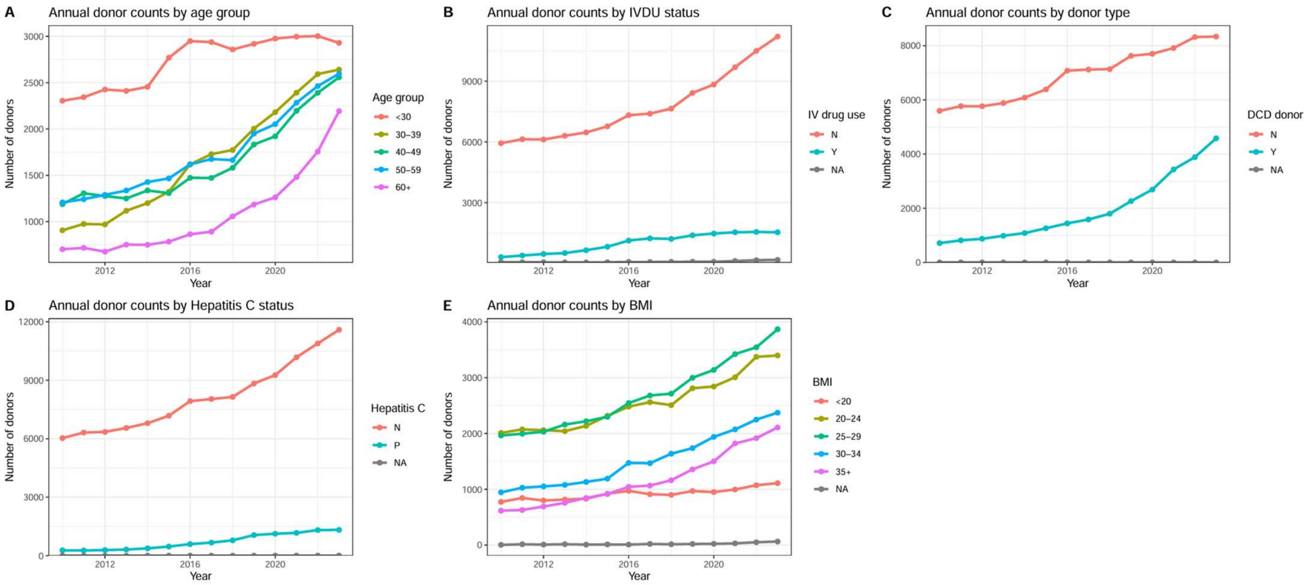

Supplementary Figure 10: Counts of key donor factors in the donors where the pancreas was used for transplant over the study period. A) Age group. B) IV drug use. C) Donor type. D) Hepatitis C status. E) BMI. 'NA' represents donors with missing data for the relevant variable. Abbreviations: IVDU = intravenous drug use, DCD = donation after circulatory death, BMI = body mass index.

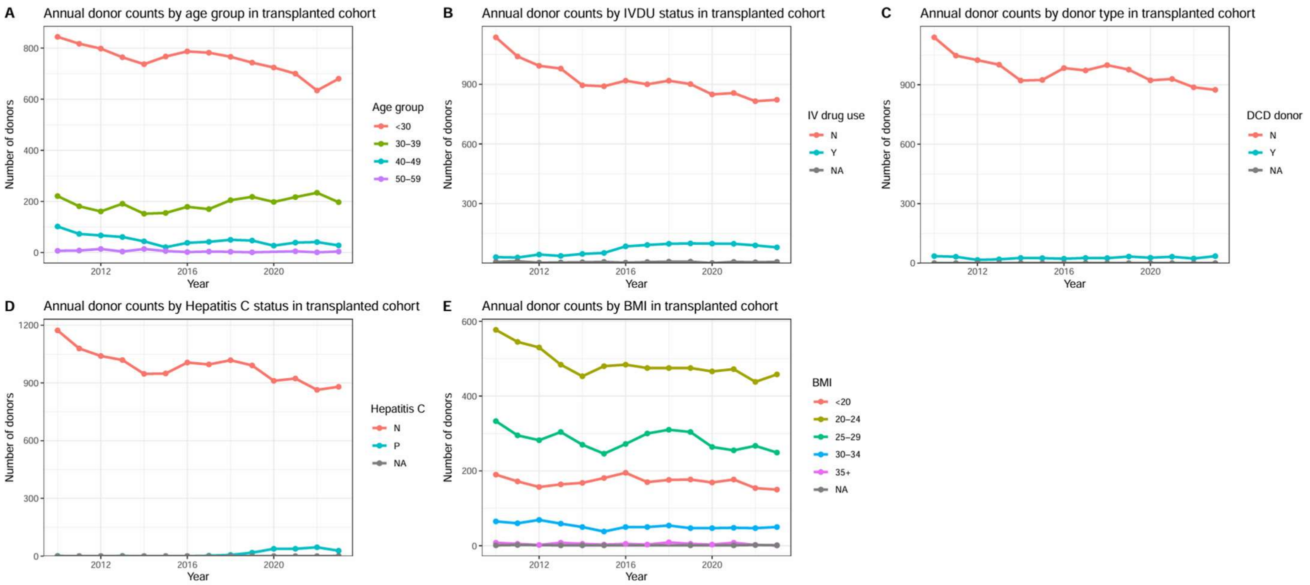

Supplement: Supplementary file 2 — Supplementary Information [file 43856_2026_1506_MOESM2_ESM.pdf]
