# Supplementary material for: Modelling donor factors influencing pancreas transplant utilization and evolution of decision-making over time
Source: Commun Med (Lond). 2026 Mar 7;6:231. doi: 10.1038/s43856-026-01506-9 (PMC13096168; doi:10.1038/s43856-026-01506-9)
Supplement: Supplementary file 3 — Supplementary Data 1 [file 43856_2026_1506_MOESM3_ESM.pdf]

**Donor characteristics**	**Pancreas not transplanted (N=119374)**	**Pancreas transplanted (N=14612)**	**Overall (N=133986)**
Age, median [IQR], y	43.0 [30.0, 55.0]	23.0 [18.0, 29.0]	40.0 [27.0, 53.0]
BMI, median [IQR], kg/m2	27.2 [23.4, 32.0]	23.4 [20.9, 26.1]	26.7 [22.9, 31.3]
Missing	315 (0.3%)	12 (0.1%)	327 (0.2%)
Ethnicity			
White	81880 (68.6%)	8932 (61.1%)	90812 (67.8%)
Asian	2778 (2.3%)	296 (2.0%)	3074 (2.3%)
Black	16881 (14.1%)	2868 (19.6%)	19749 (14.7%)
Hispanic/Latino	16265 (13.6%)	2344 (16.0%)	18609 (13.9%)
Other	1491 (1.2%)	164 (1.1%)	1655 (1.2%)
Missing	79 (0.1%)	8 (0.1%)	87 (0.1%)
Sex			
Male	71935 (60.3%)	10104 (69.1%)	82039 (61.2%)
Female	47439 (39.7%)	4508 (30.9%)	51947 (38.8%)
CMV status			
Negative	45248 (37.9%)	6034 (41.3%)	51282 (38.3%)
Positive	73329 (61.4%)	8485 (58.1%)	81814 (61.1%)
Missing	797 (0.7%)	93 (0.6%)	890 (0.7%)
Blood group			
A	44486 (37.3%)	5166 (35.4%)	49652 (37.1%)
AB	4471 (3.7%)	219 (1.5%)	4690 (3.5%)
B	13891 (11.6%)	1700 (11.6%)	15591 (11.6%)
O	56517 (47.3%)	7527 (51.5%)	64044 (47.8%)
Missing	9 (0.0%)	0 (0%)	9 (0.0%)
Cause of death			
Anoxia	34286 (28.7%)	3042 (20.8%)	37328 (27.9%)
Cerebrovascular/stroke	33776 (28.3%)	1550 (10.6%)	35326 (26.4%)
Drug overdose	16354 (13.7%)	1516 (10.4%)	17870 (13.3%)
Head trauma	30935 (25.9%)	8165 (55.9%)	39100 (29.2%)
Other	4023 (3.4%)	339 (2.3%)	4362 (3.3%)
DCD donor			
No	88286 (74.0%)	14210 (97.2%)	102496 (76.5%)
Yes	31087 (26.0%)	402 (2.8%)	31489 (23.5%)
Missing	1 (0.0%)	0 (0%)	1 (0.0%)
Given insulin 24 hours before clamp			
No insulin given	63888 (53.5%)	7178 (49.1%)	71066 (53.0%)
Insulin given	54606 (45.7%)	7367 (50.4%)	61973 (46.3%)
Missing	880 (0.7%)	67 (0.5%)	947 (0.7%)
Heavy alcohol use			
No heavy alcohol use	90315 (75.7%)	13175 (90.2%)	103490 (77.2%)
Heavy alcohol use	26696 (22.4%)	1180 (8.1%)	27876 (20.8%)
Missing	2363 (2.0%)	257 (1.8%)	2620 (2.0%)
Coronary artery disease			
No coronary artery disease	112210 (94.0%)	14568 (99.7%)	126778 (94.6%)
Coronary artery disease	6442 (5.4%)	18 (0.1%)	6460 (4.8%)
Missing	722 (0.6%)	26 (0.2%)	748 (0.6%)
Smoking			
No smoking	89391 (74.9%)	13880 (95.0%)	103271 (77.1%)

Smoking	27977 (23.4%)	615 (4.2%)	28592 (21.3%)
Missing	2006 (1.7%)	117 (0.8%)	2123 (1.6%)
Hypertension			
No hypertension	79165 (66.3%)	13996 (95.8%)	93161 (69.5%)
Hypertension	39841 (33.4%)	603 (4.1%)	40444 (30.2%)
Missing	368 (0.3%)	13 (0.1%)	381 (0.3%)
IV drug use			
No IV drug use	103929 (87.1%)	13498 (92.4%)	117427 (87.6%)
IV drug use	14236 (11.9%)	1027 (7.0%)	15263 (11.4%)
Missing	1209 (1.0%)	87 (0.6%)	1296 (1.0%)
Latest blood pH, median [IQR]	7.41 [7.36, 7.45]	7.43 [7.39, 7.46]	7.41 [7.36, 7.45]
Missing	997 (0.8%)	64 (0.4%)	1061 (0.8%)
Hepatitis C antibody status			
Negative	108632 (91.0%)	14405 (98.6%)	123037 (91.8%)
Positive	10715 (9.0%)	206 (1.4%)	10921 (8.2%)
Missing	27 (0.0%)	1 (0.0%)	28 (0.0%)
Number of rare HLA variants			
0	91844 (76.9%)	11159 (76.4%)	103003 (76.9%)
1	22477 (18.8%)	2879 (19.7%)	25356 (18.9%)
2	3223 (2.7%)	418 (2.9%)	3641 (2.7%)
3	529 (0.4%)	56 (0.4%)	585 (0.4%)
4	357 (0.3%)	24 (0.2%)	381 (0.3%)
5	535 (0.4%)	40 (0.3%)	575 (0.4%)
6	409 (0.3%)	36 (0.2%)	445 (0.3%)
Peak lipase, median [IQR], U/L	46.0 [21.0, 119]	38.0 [18.0, 92.0]	45.0 [20.0, 115]
Missing	18306 (15.3%)	68 (0.5%)	18374 (13.7%)
Peak ALT, median [IQR], U/L	83.0 [37.0, 229]	63.0 [31.0, 166]	81.0 [36.0, 221]
Missing	319 (0.3%)	1 (0.0%)	320 (0.2%)
Peak creatinine, median [IQR], U/L	1.41 [1.05, 2.13]	1.25 [1.00, 1.59]	1.40 [1.03, 2.04]
Missing	120 (0.1%)	5 (0.0%)	125 (0.1%)
Hospital stay, median [IQR], days	4.00 [3.00, 6.00]	4.00 [3.00, 6.00]	4.00 [3.00, 6.00]
Missing	583 (0.5%)	49 (0.3%)	632 (0.5%)
Inotropic support			
No inotropic support used	74831 (62.7%)	8317 (56.9%)	83148 (62.1%)
Inotropic support used	43513 (36.5%)	6210 (42.5%)	49723 (37.1%)
Missing	1030 (0.9%)	85 (0.6%)	1115 (0.8%)
