# Supplementary material for: Modelling donor factors influencing pancreas transplant utilization and evolution of decision-making over time
Source: Commun Med (Lond). 2026 Mar 7;6:231. doi: 10.1038/s43856-026-01506-9 (PMC13096168; doi:10.1038/s43856-026-01506-9)
Supplement: Supplementary file 4 — Supplementary Data 2 [file 43856_2026_1506_MOESM4_ESM.pdf]

Donor variable	Adjusted Odds Ratio (95% CI)	P-value
DCD donor	0.126 (0.095 to 0.168)	<0.001
Hepatitis C antibody positive	0.000 (0.000 to 0.000)	<0.001
Blood group		
A	Ref	
AB	0.272 (0.186 to 0.398)	<0.001
B	0.972 (0.808 to 1.169)	0.763
O	1.154 (1.022 to 1.303)	0.021
Heavy alcohol use	0.447 (0.365 to 0.547)	<0.001
IV drug use	0.247 (0.186 to 0.329)	<0.001
Ethnicity		
White	Ref	
Asian, Non-Hispanic	1.000 (0.862 to 1.160)	0.997
Black, Non-Hispanic	1.093 (1.031 to 1.159)	0.003
Hispanic/Latino	0.819 (0.771 to 0.869)	<0.001
Other	0.680 (0.564 to 0.819)	<0.001
Sex: male	0.993 (0.946 to 1.042)	0.762
CMV positive	0.905 (0.866 to 0.946)	<0.001
Cause of death		
Anoxia	Ref	
Cerebrovascular/stroke	0.974 (0.894 to 1.060)	0.537
Drug overdose	1.069 (0.985 to 1.161)	0.112
Head trauma	1.167 (1.099 to 1.240)	<0.001
Other	0.759 (0.660 to 0.873)	<0.001
Given insulin 24 hours before clamp	0.954 (0.913 to 0.997)	0.035
Coronary artery disease	0.417 (0.253 to 0.686)	<0.001
Smoking	0.735 (0.666 to 0.812)	<0.001
Hypertension	0.536 (0.486 to 0.591)	<0.001
Number of rare HLA variants		
0	Ref	
1	0.962 (0.909 to 1.018)	0.183
2	0.858 (0.722 to 1.020)	0.083
3	0.740 (0.507 to 1.079)	0.118
4	0.857 (0.490 to 1.499)	0.588
Inotropic support used	0.962 (0.920 to 1.004)	0.078
RCS: Age, years	RCS terms	<0.001
RCS: Donation date	RCS terms	<0.001
RCS: BMI	RCS terms	<0.001
RCS: Peak creatinine	RCS terms	<0.001
RCS: Peak lipase	RCS terms	<0.001
RCS: Peak ALT	RCS terms	<0.001
RCS: Latest blood pH	RCS terms	<0.001
RCS: Hospital stay, days	RCS terms	<0.001
RCS: Age, years *	RCS interaction terms	<0.001
RCS: BMI *	RCS interaction terms	0.005
RCS: DCD donor *	RCS interaction terms	0.003
RCS: Peak creatinine *	RCS interaction terms	0.004
RCS: Hepatitis C positive *	RCS interaction terms	<0.001
RCS: Blood group *	RCS interaction terms	0.898

RCS: Peak lipase *	RCS interaction terms	0.846
RCS: Peak ALT *	RCS interaction terms	<0.001
RCS: Heavy alcohol use *	RCS interaction terms	0.070
RCS: IV drug use *	RCS interaction terms	<0.001
